# Integrated time-series multi-omics reveals temporal reprogramming and inter-organ divergence in diabetic cardiorenal syndrome

**DOI:** 10.3389/fendo.2026.1950040

**Published:** 2026-10-20

**Authors:** Shan-shan Gao, Guoyue Wan, Qi Wang, Jing An, Qingmei Yang, Chuyu Liu, Yulan Cai

**Affiliations:** 1 Department of Endocrinology, The Second Affiliated Hospital of Zunyi Medical University, Zunyi, Guizhou, China; 2 Department of Biomedical Sciences, Faculty of Medicine, University of Macau, Macao, Macao SAR, China; 3 Thyroid and Breast Surgery, the Second Affiliated Hospital of Zunyi Medical University, Zunyi, Guizhou, China; 4 Department of Endocrinology and Metabolism, Affiliated Hospital of Zunyi Medical University, Zunyi, Guizhou, China

**Keywords:** cardiorenal syndrome, diabetes, ENPP2, insulin, longitudinal study, single-cell RNA-seq, time-series transcriptomics

## Abstract

Diabetes induces progressive transcriptional reprogramming in the heart and kidney, yet their tissue-specific temporal dynamics and cellular mechanisms remain incompletely understood. In this study, we employed a longitudinal design with dense sampling intervals, performing time-series bulk RNA-seq on heart and kidney tissues across six bi-weekly intervals from Control, high-fat diet (HF), diabetes (DM), and insulin-treated (INS) mice (n=38 total samples). To cross-validate and resolve cell-type-specific variations, these data were integrated with a public single-cell RNA-seq dataset (16 mouse kidneys) spanning Control, diabetic kidney disease (DKD), and SGLT2 inhibitor (SGLT2i) treatment groups. Principal component analysis and differential expression sequencing revealed a more pronounced, earlier transcriptional divergence in the kidney than in the heart under diabetic conditions. Using maSigPro regression analysis, we identified 74 core time-dependent genes in the kidney; hierarchical clustering showed that the major dynamic module was enriched in immunometabolic and inflammatory pathways. Single-cell dissection localized Enpp2 upregulation within injured proximal tubule cells (Inj.PT) of the DKD group, while MHC class I molecules (H2-K1 and H2-D1) were suppressed in immune cells. UpSet plot analysis demonstrated an overlap of these dynamic hub genes between bulk and single-cell cohorts. While insulin reversed systemic metabolic and early-phase immune aberrations in the bulk cohort, the single-cell dataset—derived from an independent SGLT2i-treated cohort—provided cellular resolution for key dynamic genes (Enpp2, H2-K1, H2-D1) and suggested that SGLT2i may similarly suppress Enpp2 in injured proximal tubule cells. These parallel observations from two distinct therapeutic interventions are hypothesis-generating and warrant direct comparative studies. These discovery-phase findings were validated by independent qPCR and immunohistochemical analyses. Taken together, this exploratory longitudinal analysis identifies tissue-specific temporal transcriptional reprogramming in diabetic heart and kidney, with evidence of partial modulation by insulin treatment.

## Graphical Abstract

**Figure:**
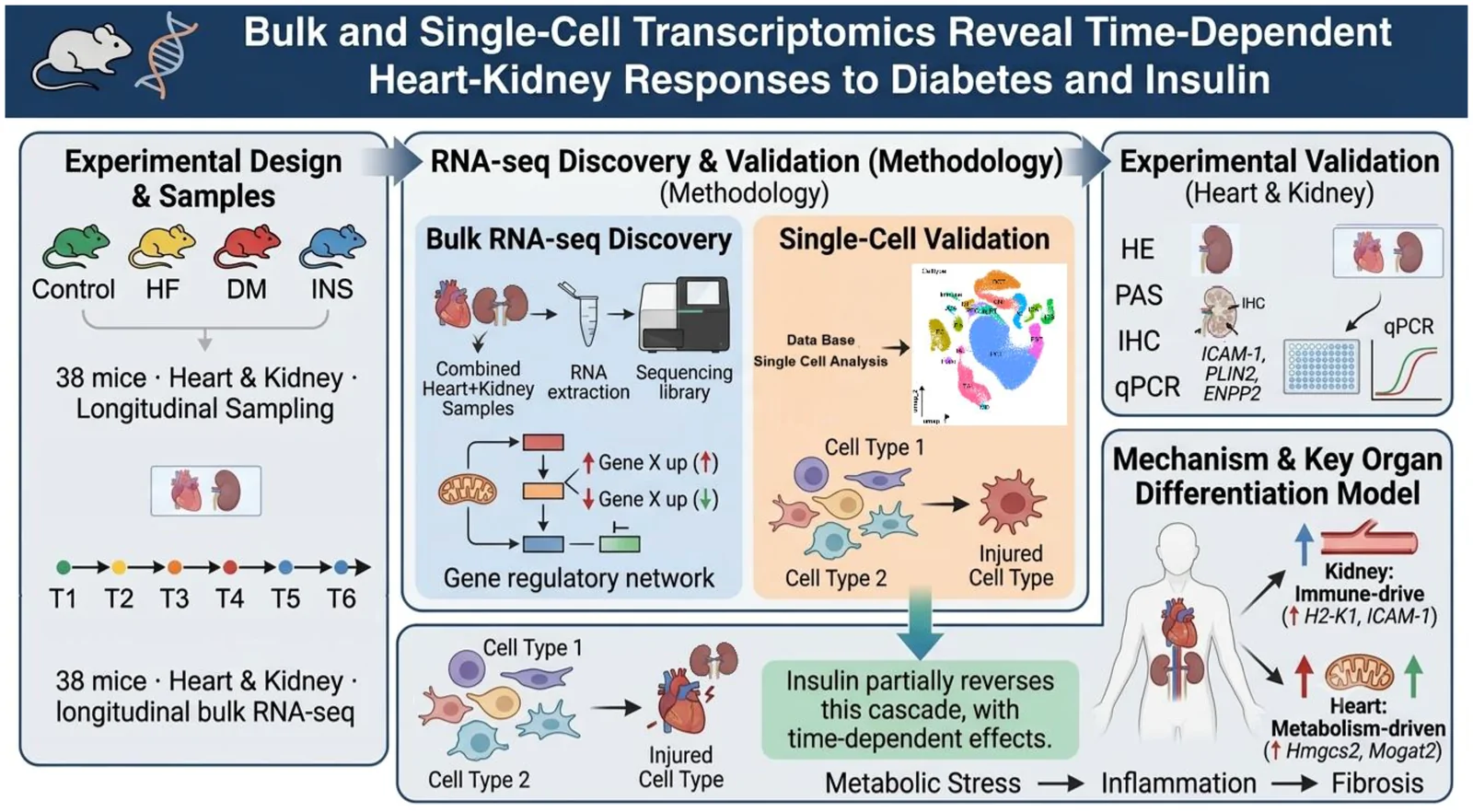

## Introduction

Diabetes mellitus and its macrovascular and microvascular complications, particularly diabetic cardiomyopathy (DCM) and diabetic kidney disease (DKD), remain major contributors to heart failure and end-stage kidney disease worldwide (1, 2). Persistent hyperglycemia, insulin resistance, lipotoxicity, and chronic inflammation place the heart and kidney under sustained metabolic stress (1, 3). These organs exhibit distinct yet interconnected injury programs within cardiorenal syndrome, underscoring the need to define shared and organ-specific mechanisms (4–6).

Guideline-directed care for diabetes and chronic kidney disease has advanced substantially. Contemporary standards recommend intensive risk-factor management together with therapies that include renin–angiotensin system blockade and sodium–glucose cotransporter-2 (SGLT2) inhibition, which improve cardiorenal outcomes (7–12). Nevertheless, residual renal and cardiovascular risk persists despite contemporary therapies, and the molecular drivers of organ-specific progression—especially their temporal dynamics—remain incompletely defined (13).

Transcriptomic studies have clarified disease-associated pathways in DCM and DKD, yet most analyses rely on single-time-point or single-organ designs and therefore cannot resolve continuous, time-dependent transcriptional reprogramming or heart–kidney divergence (14, 15). Single-cell atlases have further revealed cellular heterogeneity in the diabetic kidney, including injured proximal-tubule and immune compartments (16, 17). However, longitudinal dual-organ transcriptional trajectories, and how these trajectories respond to glycemic treatment, remain insufficiently characterized. To address this gap, we aimed to: (i) characterize temporal transcriptional divergence between the diabetic heart and kidney; (ii) examine whether time-dependent hub genes show consistent patterns across bulk and single-cell datasets; and (iii) assess whether insulin is associated with partial or module-specific modulation of these trajectories. Detailed experimental procedures are provided in the Methods; gene-level interpretation is reserved for the Results and Discussion. This work is exploratory and is not designed as a head-to-head comparison of antidiabetic drugs.

## Materials and methods

### Animal models and diabetic mouse model establishment

All animal experiments were conducted in accordance with the guidelines for laboratory animal care and were approved by the Institutional Animal Care and Use Committee/Animal Ethics Committee of Zunyi Medical University (approval No. ZMU21-2402-014). Fifty 5-week-old male C57BL/6J mice were purchased from Hunan SJA Laboratory Animal Co., Ltd. (license number: SYXK-2021-0004). Mice were housed in a specific pathogen-free (SPF) facility under controlled conditions (temperature 22 ± 2 °C, 12-h light/dark cycle) with ad libitum access to water and standard chow. After one week of acclimatization, all mice were fed a high-fat diet (HFD; XTHF60, 60% calories from fat; Synergistic Biology, Shanghai, China) for 6–8 weeks.

To establish type 2 diabetes-like conditions on the basis of high-fat diet feeding, mice were fasted overnight and then intraperitoneally injected with streptozotocin (STZ, S0130, Sigma-Aldrich, St. Louis, MO, USA) with 30 mg/kg body weight, dissolved in 0.1 M sodium citrate buffer, pH 4.5 for five consecutive days. Control mice received standard chow and were injected with an equal volume of sodium citrate buffer. One week after the final STZ injection, random blood glucose levels were measured from tail vein blood using a glucometer. Mice with random blood glucose >16.7 mmol/L (300 mg/dL) on three consecutive measurements were considered successfully induced diabetic models and included in the study.

After diabetes was established, mice were randomly assigned to four experimental groups: Control, high-fat diet (HF), diabetes (DM), and insulin-treated (INS). The DM group received no hypoglycemic intervention. For the INS group, recombinant insulin glargine injection (Changxiulin, China) was administered by subcutaneous injection into the dorsal neck region. The initial insulin dose was 0.5 U/kg, which was gradually adjusted to 0.5–1.0 U/kg based on blood glucose levels, with the goal of maintaining blood glucose within approximately 7.8–11.1 mmol/L. Blood glucose was monitored regularly, and mice with glucose levels maintained at 6.7–8.0 mmol/L were considered insulin-treated diabetic mice, when hypoglycemic risk was present, insulin administration was suspended or the dose was reduced. Insulin was selected as the in-house intervention because it is a standard clinical treatment for hyperglycemia and allowed us to test whether systemic glycemic control modulates tissue transcriptional trajectories. This study was not designed to compare insulin with metformin or SGLT2 inhibitors. SGLT2 inhibitor data were taken from an independent public single-cell dataset (GSE184652) and were used only for cell-type localization.

### Tissue collection and processing

At the start of the experiment (23 weeks of age), the DM and HF models had been established for one month prior to the baseline sampling (Time 1). For the INS group, insulin treatment was initiated at Time 2, immediately after the first sampling. To capture time-dependent transcriptomic trajectories, whole heart and kidney tissues were systematically collected across six bi-weekly intervals from Time 1 to Time 6. A total of 38 mice were utilized in the longitudinal bulk RNA-seq study. Mice were 23 weeks old at Time 1, after approximately one month of established diabetes or high-fat feeding. The six visits therefore span about 10 weeks. This window was chosen to capture early-to-intermediate transcriptional change in the HFD/STZ model, not end-stage human cardiorenal disease.

At the end of the experiment, mice were anesthetized and euthanized according to the approved protocol. Heart and bilateral kidney tissues were collected, rinsed with cold PBS, and dried. Part of each tissue was fixed in 4% paraformaldehyde for histological and immunohistochemical analyses, while the remaining tissue was snap-frozen in liquid nitrogen and stored at −80 °C for molecular assays. All surgical and sampling procedures were performed under appropriate anesthesia, and every effort was made to minimize animal suffering.

### Bulk RNA sequencing and principal component analysis

Total RNA was extracted from heart and kidney tissues using TRIzol reagent (Thermo Fisher Scientific, Cat. No. 15596018) according to the manufacturer’s instructions. High-throughput time-series bulk RNA sequencing (RNA-seq) was performed 2×150bp paired-end sequencing (PE150) on an Illumina Novaseq™ X Plus (LC-Bio Technology CO., Ltd., Hangzhou, China) following the vendor’s recommended protocol. Raw reads were quality-controlled using FastQC and mapped to the mouse reference genome mm10 using Hisat2. Principal Component Analysis (PCA) was applied to the normalized gene expression matrices of the heart and kidney datasets independently. PCA plots were generated using the top principal components (PC1 and PC2) to visualize and compare the global transcriptomic clustering patterns and trajectory paths of the four experimental groups over the six time points. The discovery bulk RNA-seq cohort used one animal per group per time point (38 samples in total). Control and HF covered six time points. DM covered four time points, and INS covered three time points because insulin was started at Time 2. Exact numbers are listed in **Supplementary Table S1**. PCA was used as a descriptive visualization.

### Differential expression and functional enrichment analysis

Differentially expressed genes (DEGs) were calculated across major experimental group comparisons using the edgeR R package (18). The thresholds for identifying significant DEGs were set at |log2FC| > 1, adj.P < 0.05. Overlapping and unique DEGs among the different contrast groups were visualized using Venn diagrams for both heart and kidney tissues. For functional annotation, the DEGs from the renal DM vs. Control comparison were subjected to enrichment analysis. The enrichment was performed using Gene Ontology Biological Process (GO BP) terms via clusterProfiler (19). Significantly enriched GO CC categories were filtered based on a cut-off threshold of *adj.P* < 0.05 and displayed via a bubble plot based on gene counts.

### Time-dependent profile analysis (maSigPro)

To systematically identify genes exhibiting significant time-dependent expression patterns, the regression-based approach in the maSigPro R package was executed (20). maSigPro analysis was applied to both the kidney and heart datasets across the major group contrasts (DM vs. Control, HF vs. Control, and INS vs. Control) using a significance threshold of Q <0.3 and an R-squared threshold of R2 >0.6. Given the discovery-oriented nature of the longitudinal screening cohort (n = 1 per group per timepoint), all maSigPro-identified genes were considered exploratory and subjected to independent validation by qPCR (n = 3 per group per timepoint) and immunohistochemistry (N = 15 per group). Core time-dependent renal genes were defined as maSigPro-significant genes (Q < 0.3, R² > 0.6) retained as the shared or core set across HF vs Control, DM vs Control, and INS vs Control. This yielded 74 unique genes. Ensembl IDs, gene symbols and contrast membership are provided in **Supplementary Table S2**. Given the discovery design (n = 1 per group per time point), these genes were treated as exploratory and were checked by independent qPCR and immunohistochemistry. These genes were grouped into functional categories including Fibrosis, Inflammation, and Metabolism.

The identified time-dependent renal genes were further partitioned into four distinct clusters using regression profile modeling based on their temporal trajectories across the six time points. Individual regression curve-fitting plots were constructed for representative hub candidates to track group-specific temporal changes. Protein-protein interaction (PPI) networks for specific clusters were retrieved from the STRING database (21) and visualized to highlight core interconnected gene nodes. For the cardiac dataset, a functionally grouped network of enriched pathways was constructed from its time-dependent genes to map tissue-specific modules.

### Public single-cell RNA-seq data processing

For single-cell level validation, a public single-cell RNA sequencing (scRNA-seq) dataset derived from mouse kidneys was obtained from the GEO database under the accession number GSE184652 (22). This validation dataset comprised sequencing data from a total of 16 mice, which were distributed across three experimental frameworks: Control, diabetic kidney disease (DKD), and Treatment groups (**Figure 1B**). The single-cell expression matrix was analyzed using the Seurat v5 R package (23).

**Figure 1:**
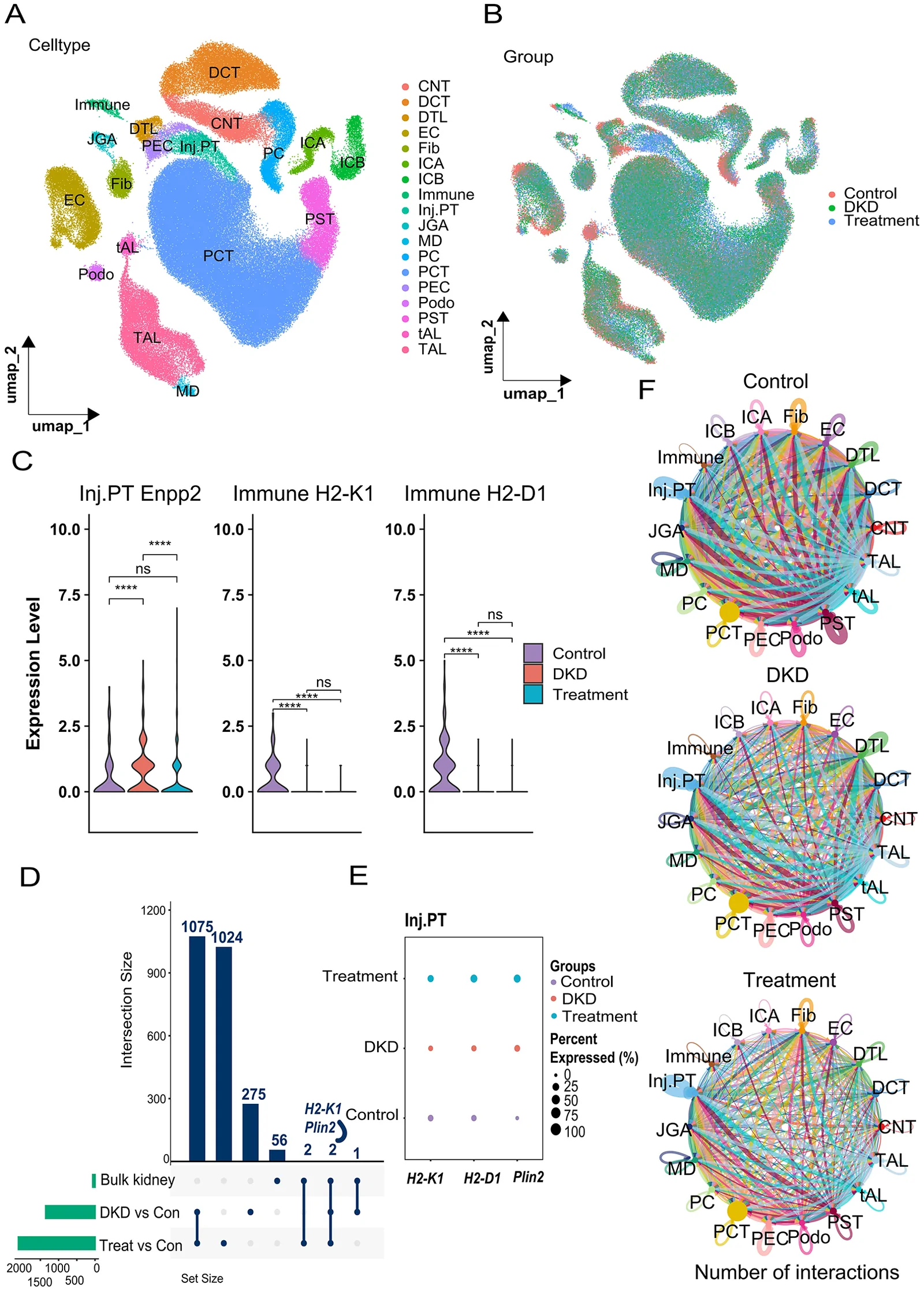
Single-cell RNA-seq provides additional cellular resolution of key gene changes in diabetic kidney. **(A)** UMAP visualization of 18 annotated major renal cell types. **(B)** UMAP plots colored by experimental group (Control, DKD, Treatment). **(C)** Violin plots showing expression levels of Enpp2 in injured proximal tubule cells (Inj.PT) and H2-K1, H2-D1 in immune cells across groups. **(D)** UpSet plot demonstrating the overlap between the 74 maSigPro time-dependent genes from bulk kidney RNA-seq and differentially expressed genes in Inj.PT cells (DKD vs Control and Treatment vs Control). **(E)** Dot plot showing the expression proportion of selected genes (H2-K1, H2-D1, Plin2) in Inj.PT cells across groups. **(F)** CellChat circle plots showing cell-cell communication networks in Control, DKD, and Treatment groups. Line thickness represents interaction strength.

Cells with fewer than 200 detected genes or a mitochondrial gene percentage greater than 5% were excluded. Cell clusters were visualized using the uniform manifold approximation and projection (UMAP) embedding method to resolve major renal cell lineages. Cell types were defined based on established canonical markers, identifying proximal convoluted tubules (PCT), proximal straight tubules (PST), injured proximal tubules (Inj.PT), podocytes (Podo), endothelial cells (EC), fibroblast cells (Fib), and immune cells (Immune) (**Figure 1A**). The specific cell-type expression levels of selected marker genes (Enpp2, H2-K1, and H2-D1) were evaluated across the three groups via violin plots. Statistical significance among groups was calculated using the non-parametric Wilcoxon rank-sum test, with significance defined as *P < 0.05*.

### Cell-cell communication analysis

To analyze multicellular signaling interactions within the renal microenvironment, cell-cell communication networks were inferred using the CellChat R package (24). CellChat was applied separately to the single-cell datasets of the Control, DKD, and Treatment groups based on the CellChatDB.mouse ligand-receptor interaction database. The total number of cellular interactions and the overall interaction frequencies among the resolved cell lineages were calculated and visualized as circle network graphs for each group.

To quantify the roles of individual cell types, the incoming and outgoing interaction strengths were measured for each lineage. These metric values were mapped onto two-dimensional scatter plots for the DKD and Treatment networks, allowing the comparative tracking of coordinate shifts for functional, interstitial, and injured epithelial components (such as Inj.PT, Podo, and Fib) within the intercellular signaling space.

### Quantitative real-time PCR

Total RNA was extracted from heart and kidney tissues using MolPure^®^ Mag16/48 Tissue/Cell Total RNA Kit (18607ES16, Yeasen, China) according to the manufacturer’s protocol. Heart and kidney tissues were harvested from three independent mice per group at each time point, representing three independent biological replicates. RNA concentration and purity were determined using a NanoDrop spectrophotometer (Thermo NanoDrop ONE, Thermo Fisher, USA). cDNA was synthesized from 1 μg of total RNA using the Hifair^®^ AdvanceFast 1st Strand cDNA Synthesis SuperMix for qPCR (11155ES60, Yeasen, China) following the manufacturer’s instructions.

qPCR was performed on a Qtower Real-Time PCR System (Qtower, Germany) using NovoStart^®^ Universal Fast SYBR qPCR SuperMix (E401-01A, Novoprotein, Shanghai, China). The PCR conditions were as follows: 95 °C for 30 s, followed by 40 cycles of 95 °C for 5 s and 60 °C for 30 s.

The primer sequences used were as follows:

H2-K1: forward 5’- TGACCTGGCAGTTGAATG -3’, reverse 5’- GTTGGAGACAGTGGATGG -3’.
H2-D1: forward 5’- GGAAGGAGCAGAATTACACA -3’, reverse 5’- TGGAGCCAGAGCATAGTC -3’.
Enpp2: forward 5’- GATTACAGCCACCAAGCA -3’, reverse 5’- GCCATAACTACTCTCCTCAG -3’.
Plin2: forward 5’- GAACAGTGGAGTAGATAATGC -3’, reverse 5’- CTGAGAGCCTGGTGATAAG -3’.
Icam1: forward 5’- GGAATGTCACCAGGAATGT -3’, reverse 5’- CTGAGCCTTCTGTAACTTGT -3’.
Hmgcs2: forward 5’-AGCCTACCGCAAGAAGAT -3’, reverse 5’- GACAGGAAGTCATTGAACATC -3’.
Mogat2: forward 5’- ATCCGCTCCTATCTGATGA -3’, reverse 5’- GATGATGGCAAGCAAGTTC -3’
β-actin (internal control): forward 5’- GCACCACACCTTCTACAA -3’, reverse 5’- TACGACCAGAGGCATACA -3’.

The relative mRNA expression levels were calculated using the 2^-^ΔΔCt method and normalized to β-actin as the internal control. All reactions were performed in triplicate, and the average Ct values were used for analysis.

### Histological and immunohistochemical staining

Kidney tissues were fixed in 4% paraformaldehyde, embedded in paraffin, and sectioned at 3 μm thickness. For histological and immunohistochemical analysis, kidney tissues were collected from two independent mice per group (**Supplementary Table S1**). For histopathological evaluation, sections were stained with hematoxylin and eosin (HE) for assessment of overall renal morphology. Briefly, sections were dewaxed, hydrated, treated with HD constant staining pretreatment solution for 1 min, stained with hematoxylin for 3–5 min, differentiated, blued, and then stained with eosin for 15 s. For evaluation of glomerular basement membrane (GBM) thickening and mesangial matrix expansion, periodic acid-Schiff (PAS) staining was performed according to the manufacturerprotocol. Briefly, sections were oxidized with PAS Solution B for 15 min, followed by Schiff reagent (PAS Solution A) for 30 min in the dark, and counterstained with PAS Solution C for 30 s.

For immunohistochemical staining, sections were deparaffinized, rehydrated, and subjected to antigen retrieval in EDTA buffer (pH 8.0) using a pressure-heating antigen retrieval apparatus at 100 °C for 15 min. Endogenous peroxidase activity was blocked with 3% methanol-H_2_O_2_ for 25 min at room temperature in the dark. Sections were blocked with 3% BSA for 30 min at room temperature, then incubated with primary antibodies overnight at 4 °C. The following primary antibodies were used: anti-ICAM1 (1:800, HA723878, Huabio, China), anti-PLIN2 (1:200, ET1704-17, Huabio, China), and anti-ENPP2 (1:200, K111498P, Solarbio, China). After washing with PBS, sections were incubated with HRP-conjugated secondary antibody (S-vision multi-polymer detection system, G1302, Solarbio, China) for 50 min at room temperature. Signals were visualized using DAB substrate, and sections were counterstained with hematoxylin. All sections were examined and photographed under a light microscope (Nikon E100) at ×20 and ×40 magnification.

### Image acquisition and quantitative analysis

For each stained section, digital images were captured using a confocal laser scanning microscope (A1R, Nikon, Japan) and analyzed. From each section, 15 representative fields were randomly selected at ×20 magnification, evenly distributed across the cortical region to ensure comprehensive coverage of glomerular and tubular areas. Each image was cropped to a uniform size of 1000 × 1000 pixels (or 500 × 500 pixels) using Saiviewer^®^ digital slide browsing software (Servicebio, China) for standardized analysis.

For immunohistochemical staining, H-score was calculated as the primary quantitative parameter. Briefly, staining intensity was graded on a scale of 0 to 3 (0, negative; 1, weak positive [light yellow]; 2, moderate positive [yellow-brown]; 3, strong positive [brown]), and the percentage of cells at each intensity level was determined within the cropped fields. The H-score was calculated using the formula: H-score = Σ (intensity score × percentage of cells at that intensity), yielding a possible range of 0 to 300. For ICAM1, which is predominantly membrane-localized, the percentage of membrane-positive cells at each intensity level was evaluated. For PLIN2 and ENPP2, which are predominantly cytoplasmic, the percentage of cytoplasmic-positive cells at each intensity level was evaluated. All quantitative analyses were performed using ImageJ software and Saiviewer^®^ measurement tools. All evaluations were performed by two independent observers blinded to the experimental groups, and the average values were used for statistical analysis.

### Data analysis

All experiments were performed at least in triplicate, analyzed by PRISM GraphPad software. Data are presented as mean ± Standard Error of Mean (SEM). Statistical significance was determined using one-way or two-way ANOVA followed by Tukey’s *post hoc* test. A p-value of less than 0.05 was considered statistically significant. (* P < 0.05,  ** P < 0.01,  *** P < 0.001,  **** P < 0.0001).

### Data availability

The raw sequence data reported in this paper have been deposited in the Genome Sequence Archive (GSA: CRA047585) at the National Genomics Data Center, China National Center for Bioinformation/Beijing Institute of Genomics, Chinese Academy of Sciences, and are publicly accessible at https://ngdc.cncb.ac.cn/gsa. The public single-cell RNA-seq dataset used in this study is available in the GEO database under accession number GSE184652. All other supporting data are included in the article and its **Supplementary Material** (**Supplementary Table S1**, **S2**).

## Result

### Global transcriptomic profiles in heart and kidney under metabolic stress

We performed time-series bulk RNA-seq on heart and kidney tissues. To capture continuous temporal trends, we sampled one animal per group at each of the six bi-weekly intervals. The study included 38 samples in total. Control and HF groups covered all six timepoints. The DM group covered four timepoints after model establishment and RNA quality control, the INS group covered three timepoints because insulin was started at Time 2 (**Figure 2A**; **Supplementary Table S1**). Principal component analysis was used as a descriptive visualization of this discovery cohort (**Figure 2A**).

**Figure 2:**
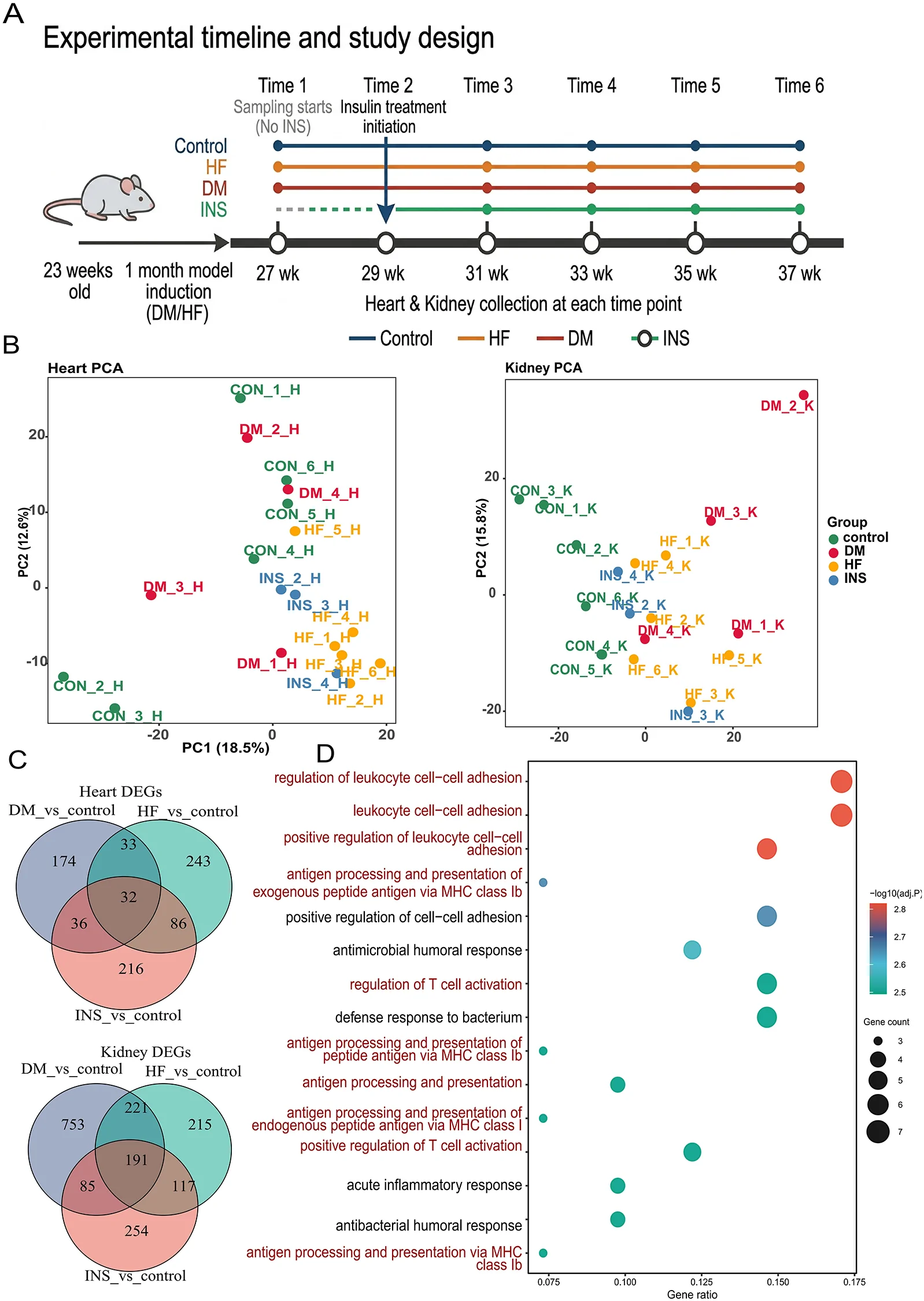
Experimental design and global transcriptomic profiling of heart and kidney in response to diabetes, high-fat diet, and insulin treatment. The longitudinal RNA-seq data were analyzed as a discovery cohort to identify temporal transcriptional patterns, with subsequent independent validation by qPCR and immunohistochemistry. **(A)** Schematic timeline of the experimental design. Mice were 23 weeks old at the beginning of the study. DM and HF models were induced for 1 month prior to the first sampling (Time 1). Insulin treatment was initiated from Time 2. Heart and kidney tissues were collected every two weeks up to Time 6. **(B)** Principal component analysis (PCA) of RNA-seq data from heart (left) and kidney (right) tissues, colored by experimental group. **(C)** Venn diagrams showing the overlap of differentially expressed genes (DEGs) in heart (upper) and kidney (lower) across indicated comparisons. **(D)** Bubble plot of significantly enriched GO Biological Process (BP) terms for kidney DEGs (DM vs Control). Bubble size represents gene count and color represents -log10(adjusted P value).

Principal component analysis (PCA) showed different clustering patterns for each organ (**Figure 2B**). In the heart, Control samples clustered together, while DM samples shifted along PC1 over time. HF samples occupied an intermediate position. INS samples were located between the Control and DM clusters. In the kidney, the distance between groups was larger than in the heart. DM samples showed a higher spatial deviation from the Control cluster (**Figure 2B**).

We next identified differentially expressed genes (DEGs; |log_2_FC| > 1, adj. P < 0.05). The kidney had more DEGs than the heart across the evaluated contrasts (**Figure 2C**). Venn diagrams showed that the DM versus Control contrast had the most unique changes in the kidney. Part of the DM-induced DEGs overlapped with HF-induced changes in both organs. Insulin treatment partially attenuated a subset of these transcriptional changes (**Figure 2C**). To further characterize the biological processes associated with these renal DEGs, we performed GO Biological Process (BP) enrichment analysis. This confirmed significant enrichment in antigen processing and presentation via MHC class I and II, inflammatory response, and leukocyte activation (FDR < 0.05), corroborating the interpretation of immunological remodeling in the diabetic kidney (**Figure 2D**). These results indicate that metabolic stress induces immune and inflammatory remodeling in the kidney.

### Time-dependent gene expression patterns identified by maSigPro

We applied maSigPro analysis to identify time-dependent genes. The analysis focused on consistent temporal trends across the sampling period. The kidney contained more time-dependent genes than the heart in the DM versus Control and INS versus DM contrasts (**Figure 3A**).

**Figure 3:**
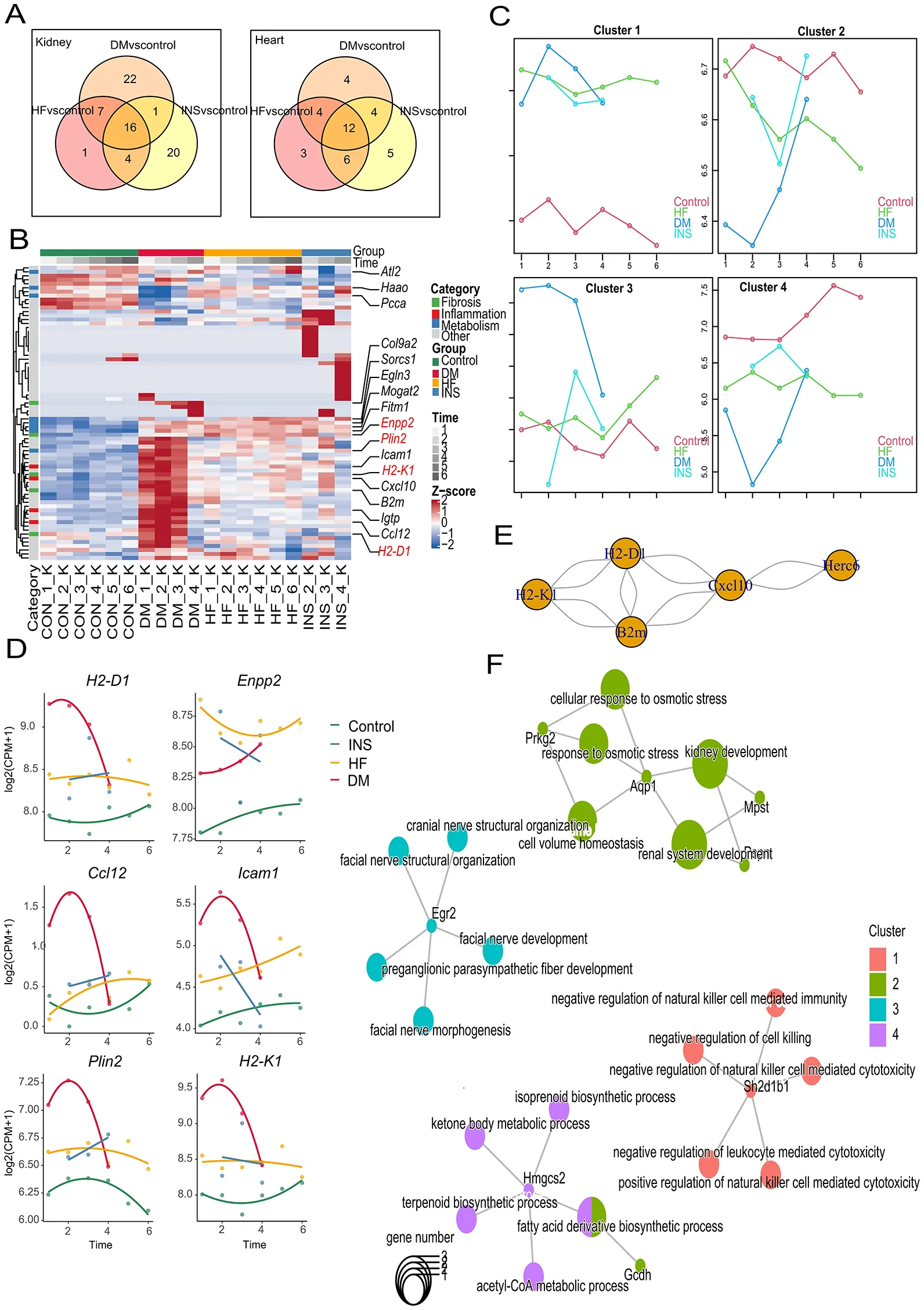
Temporal expression patterns identified by maSigPro in heart and kidney tissues. **(A)** Number of time-dependent genes identified by maSigPro in different contrasts for kidney (left) and heart (right). **(B)** Heatmap of the 74 core time-dependent genes in kidney across groups and time points. **(C)** Average expression trajectories of the four gene clusters in kidney. **(D)** Representative individual gene trajectories with maSigPro fitted curves. **(E)** PPI network of Cluster 3 genes in kidney. **(F)** Functionally grouped network of enriched pathways for time-dependent genes in heart.

In the kidney, 74 unique core time-dependent genes were retained as the shared or core maSigPro set across the major renal contrasts (Q < 0.3, R² > 0.6; **Supplementary Table S2**). Hierarchical clustering divided these genes into four distinct expression clusters (**Figures 3B, C**). Cluster 3 was the largest cluster and contained immune-related genes. These genes included *Cxcl10*, *Icam1*, *B2m*, *H2-K1*, and *H2-D1*. Cluster 1 contained metabolism-related genes, such as *Mogat2* and *Enpp2*.

Trajectory plots showed temporal alterations in the DM group (**Figure 3D**). Several inflammatory genes showed an early increase followed by a decline. Insulin treatment partially altered these temporal trajectories within specific time windows. Protein-protein interaction (PPI) network analysis of Cluster 3 identified *H2-D1*, *H2-K1*, *B2m*, and *Cxcl10* as central nodes (**Figure 3E**).

In the heart, time-dependent genes were enriched in functionally distinct pathways compared to the kidney. GO Biological Process enrichment analysis of the 22 cardiac time-dependent genes revealed enrichment in both immune-related and metabolic pathways (**Table 1**). Immune-related terms included *leukocyte mediated cytotoxicity* (GO:0001909; FDR = 0.0059) and *T cell mediated cytotoxicity* (GO:0001913; FDR = 0.0070), with key genes including *Prf1*, *Gzmb*, *Fas*, and *Cd8a*, suggesting a component of immune activation in the diabetic heart. Concurrently, metabolic pathways were also enriched, including *fatty acid derivative biosynthetic process* (GO:1901570; FDR = 0.0136), *acyl-CoA metabolic process* (GO:0006637; FDR = 0.0568), and *ketone body metabolic process* (GO:1902224; nominal *P* = 0.0077). Key metabolic genes identified included *Hmgcs2* (ketogenesis), *Gcdh* (fatty acid oxidation), and *Scd1* (lipid desaturation). In contrast to the kidney, where immune-related pathways dominated the temporal transcriptional response (**Figures 3B, C**), the cardiac time-dependent genes exhibited a mixed profile with a prominent metabolic adaptation component, highlighting tissue-specific divergence in the transcriptional response to systemic metabolic stress (**Figure 3F**). These results show tissue-specific temporal profiles between the heart and the kidney under diabetic conditions.

**Table 1:** GO biological process enrichment analysis of cardiac time-dependent genes.

GO ID	Term	Count	FDR	Genes (symbol)
GO:0001909	leukocyte mediated cytotoxicity	4	0.0059	Prf1, Gzmb, Fas, Cd8a
GO:0001913	T cell mediated cytotoxicity	3	0.007	Prf1, Gzmb, Cd8a
GO:1901570	fatty acid derivative biosynthetic process	2	0.0136	Gcdh, Hmgcs2
GO:0002449	lymphocyte mediated immunity	4	0.0195	Prf1, Gzmb, Fas, Cd8a
GO:0002456	T cell mediated immunity	3	0.0206	Prf1, Gzmb, Cd8a
GO:0006884	cell volume homeostasis	2	0.0206	Slc12a2, Aqp2
GO:0008366	axon ensheathment	3	0.0206	Mbp, Egr2, Gzmb
GO:0007272	ensheathment of neurons	3	0.0206	Mbp, Egr2, Gzmb
GO:0006970	response to osmotic stress	2	0.0575	Slc12a2, Aqp2
GO:0006637	acyl-CoA metabolic process	2	0.0568	Gcdh, Hmgcs2
GO:0033539	fatty acid beta-oxidation using acyl-CoA dehydrogenase	1	0.0808	Gcdh
GO:1902224	ketone body metabolic process	1	0.0808	Hmgcs2
GO:0006635	fatty acid beta-oxidation	1	0.1152	Gcdh

### Independent single-cell validation of key dynamic genes in the diabetic kidney

We integrated the bulk time-series data with a public single-cell RNA-seq (scRNA-seq) dataset of 16 mouse kidneys to resolve cellular heterogeneity. This independent cohort, which included SGLT2 inhibitor (SGLT2i) treatment as a comparator, was used primarily to validate the cell-type-specific expression patterns of key dynamic genes identified in our bulk discovery cohort, rather than to directly compare therapeutic efficacy between insulin and SGLT2i. This independent cohort contained Control, diabetic kidney disease (DKD), and SGLT2i treatment groups. Uniform Manifold Approximation and Projection (UMAP) identified 18 distinct renal cell types (**Figures 1A, B**). Cellular expression analysis showed that *Enpp2* expression was significantly higher in injured proximal tubule cells (Inj.PT) of the DKD group than the Control group (**Figure 1C**, *P<0.001*). SGLT2i treatment was associated with lower *Enpp2* expression in Inj.PT cells (**Figure 1C**). MHC class I transcripts *H2-K1* and *H2-D1* were lower in immune cells of the DKD group than in controls (**Figure 1C**). The DKD versus SGLT2i contrast for *H2-K1* and *H2-D1* was not uniformly significant. UpSet plot analysis demonstrated an overlap between the 74 time-dependent renal genes and the cell-type-specific differentially expressed genes (**Figure 1D**). Specifically, *H2-K1* and *Plin2* were altered across both datasets. In Inj.PT cells, the proportion of cells expressing *H2-K1* and *H2-D1* was lower in the DKD group; the SGLT2i group showed a higher proportion than DKD, but this contrast should be interpreted with caution (**Figure 1E**).

CellChat analysis suggested altered cell-cell communication networks in the DKD group, characterized by increased signaling strength involving Inj.PT and reduced interactions associated with fibroblasts (Fib) (**Figure 1F**). These patterns were partially shifted in the SGLT2i group. These scRNA-seq findings show cell-type-specific variations in metabolic and immune markers under diabetic conditions and therapeutic intervention.

### Validation of dynamic gene trajectories and inter-organ correlation analysis

To validate the bulk RNA-seq findings, we performed RT-qPCR analysis on selected hub genes using an independent cohort (n=3 mice per group per timepoint) at timepoints 2, 3, and 4 (**Supplementary Table S1**). In the heart, the metabolic transcripts *Mogat2* and *Hmgcs2* displayed time-dependent variations (**Figure 4A**). For *Mogat2*, expression levels significantly increased in both HF and DM groups at timepoint 2 (*P<0.001*), while insulin treatment partially attenuated this upregulation at timepoint 2 with a delay upregulation at timepoint 3, and dropping dramatically at timepoint 4 (*P<0.001*). For *Hmgcs2*, a statistically significant elevation was observed in the DM group from timepoint 2 (*P<0.001*), which was altered under insulin intervention at timepoints 2, 3 and 4 (*P<0.001*).

**Figure 4:**
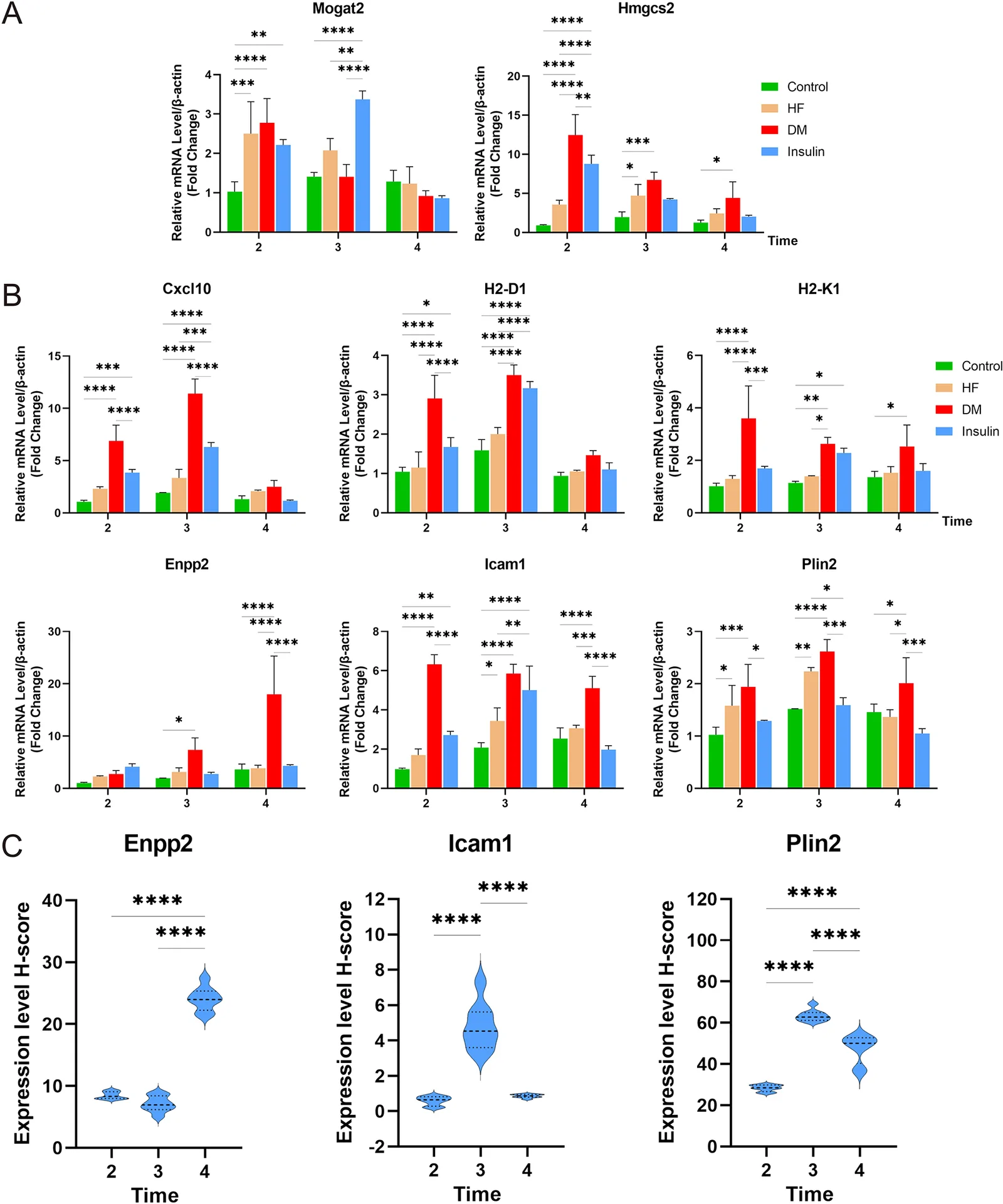
qPCR validation of cardiac and renal genes, and IHC temporal profiling of insulin-treated group. **(A)** Relative mRNA expression levels of cardiac metabolic genes Mogat2 and Hmgcs2 in heart tissues at Time points 2, 3, and 4 across the four experimental groups (Control, HF, DM, and INS). **(B)** Relative mRNA expression levels of renal immune-inflammatory and fibrosis-related genes (*Enpp2*, *H2-K1*, *H2-D1*, *Cxcl10*, *Icam1*, and *Plin2*) in kidney tissues at Time points 2, 3, and 4 across the four experimental groups. For qPCR, data were obtained from three independent mice per group, with each sample run in triplicate (n = 3 biological replicates, each with 3 technical replicates). **(C)** Violin plots with overlaid scatter points showing H-score temporal dynamics of ENPP2, ICAM1, and PLIN2 immunohistochemical staining in the INS group at Time points 2, 3, and 4. Each dot represents the H-score from one randomly captured field (n = 8 fields per time point, randomly sampled across sections of the INS group). Data are presented as mean ± SEM. Statistical significance was determined by two-way ANOVA followed by Tukey’s *post hoc* test. *p < 0.05, **p < 0.01, ***p < 0.001; ns, not significant.

In the kidney, immune-related genes showed early-phase activation. Compared to the Control group, *Cxcl10*, *H2-D1*, and *H2-K1* expression levels increased significantly in the DM group at timepoints 2 and 3 (**Figure 4B**, *P<0.0001*). This elevation declined at timepoint 4. Insulin intervention significantly suppressed the expression of these three immune markers across specific intervals (*P<0.05*). Conversely, lipid- and fibrosis-related transcripts displayed late-phase accumulation trends. *Enpp2* expression remained stable early but increased significantly in the DM group at timepoint 4 (*P<0.0001*). *Icam1* and *Plin2* expression levels were persistently higher in the DM group across the evaluated intervals (*P<0.01*). Insulin treatment consistently decreased the expression profiles of these dynamic transcripts in the kidney (*P<0.05*).

At the protein level, immunohistochemical quantitative H-scores for ENPP2, ICAM1, and PLIN2 in the insulin-treated group showed distinct temporal patterns (**Figure 4C**). The protein level of ENPP2 remained unchanged between timepoints 2 and 3, followed by a significant increase at timepoint 4 (*P<0.0001*). In contrast, the H-scores for ICAM1 and PLIN2 increased significantly from timepoint 2 to timepoint 3 (*P<0.0001*), and then decreased at timepoint 4 (*P<0.0001*). These findings show that diabetes induces tissue-specific, time-dependent transcriptomic variations, which are partially modulated under systemic insulin intervention.

### Insulin intervention attenuates diabetes-induced renal histopathology and protein expression alterations

To evaluate structural changes and protein-level translation in the kidney, we performed histological staining and quantitative immunohistochemistry (IHC) across the Control, HF, DM, and INS groups (**Figure 5**). Hematoxylin and eosin (HE) along with Periodic acid–Schiff (PAS) staining revealed localized structural variations in the renal cortex (**Figure 5A**). The DM group demonstrated alterations in glomerular and tubular morphology, whereas the INS group showed structural profiles closer to the Control group (**Figure 5A**). Quantitative analysis of IHC H-scores was performed using 15 randomly captured fields per group (N = 15) for three dynamic markers: *Enpp2*, *Plin2*, and *Icam1* (**Figures 5B, C**). For *Enpp2*, H-scores significantly increased in the HF group compared to the Control group (*P* < 0.0001), but not significantly changed in the DM group and reached the lowest levels in the INS group (*P* < 0.0001). Importantly, IHC analysis revealed that ENPP2 protein was predominantly localized to injured proximal tubular epithelial cells (Inj.PT) in the DM group, and insulin treatment markedly reduced this expression (**Figure 5C**). For *Plin2*, the expression levels increased in the DM group compared to both Control (*P* < 0.01) and HF groups, while insulin treatment significantly suppressed this elevation (*P* < 0.0001). For *Icam1*, expression levels increased significantly in the DM group, which was significantly attenuated under insulin intervention (*P* < 0.0001). Interestingly, ICAM1 expression in the HF group was unexpectedly lower than that in the Control group, raising the possibility that high-fat feeding alone may transiently suppress specific renal inflammatory pathways at this early stage, a finding that merits further mechanistic exploration. These findings demonstrate that insulin treatment alters diabetes-induced histological remodeling and protein-level expression patterns in the kidney (**Figure 6**).

**Figure 5:**
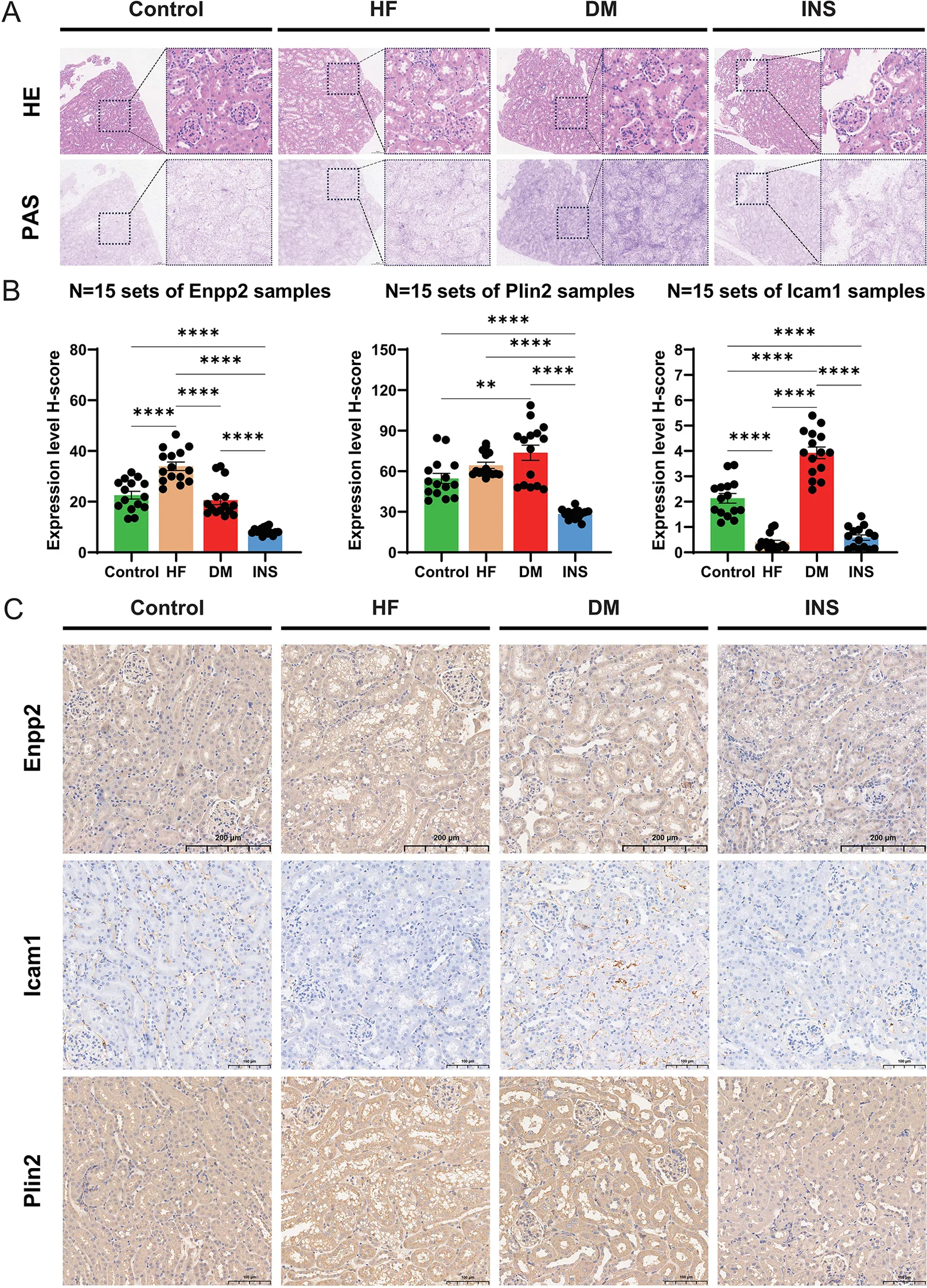
Histopathological assessment and immunohistochemical characterization of kidney tissues at Time point 2. **(A)** Representative HE and PAS staining images showing glomerular morphology across the four experimental groups (Control, HF, DM, and INS). Scale bar = 100 μm. **(B)** Quantitative analysis of IHC staining using H-score for ENPP2, ICAM1, and PLIN2 across the four groups. Each dot represents the H-score from one randomly captured field (n = 15 randomly captured fields per group). Data are presented as mean ± SEM. **(C)** Representative IHC staining images of ENPP2, ICAM1, and PLIN2 in kidney sections from each group. Positive signals are indicated by brown DAB staining; nuclei were counterstained with hematoxylin. Scale bars: ENPP2, 200 μm; ICAM1 and PLIN2, 100 μm. Statistical significance was determined by Two-way ANOVA followed by Tukey’s *post hoc* test. *p < 0.05, **p < 0.01, ***p < 0.001; ns, not significant.

**Figure 6:**
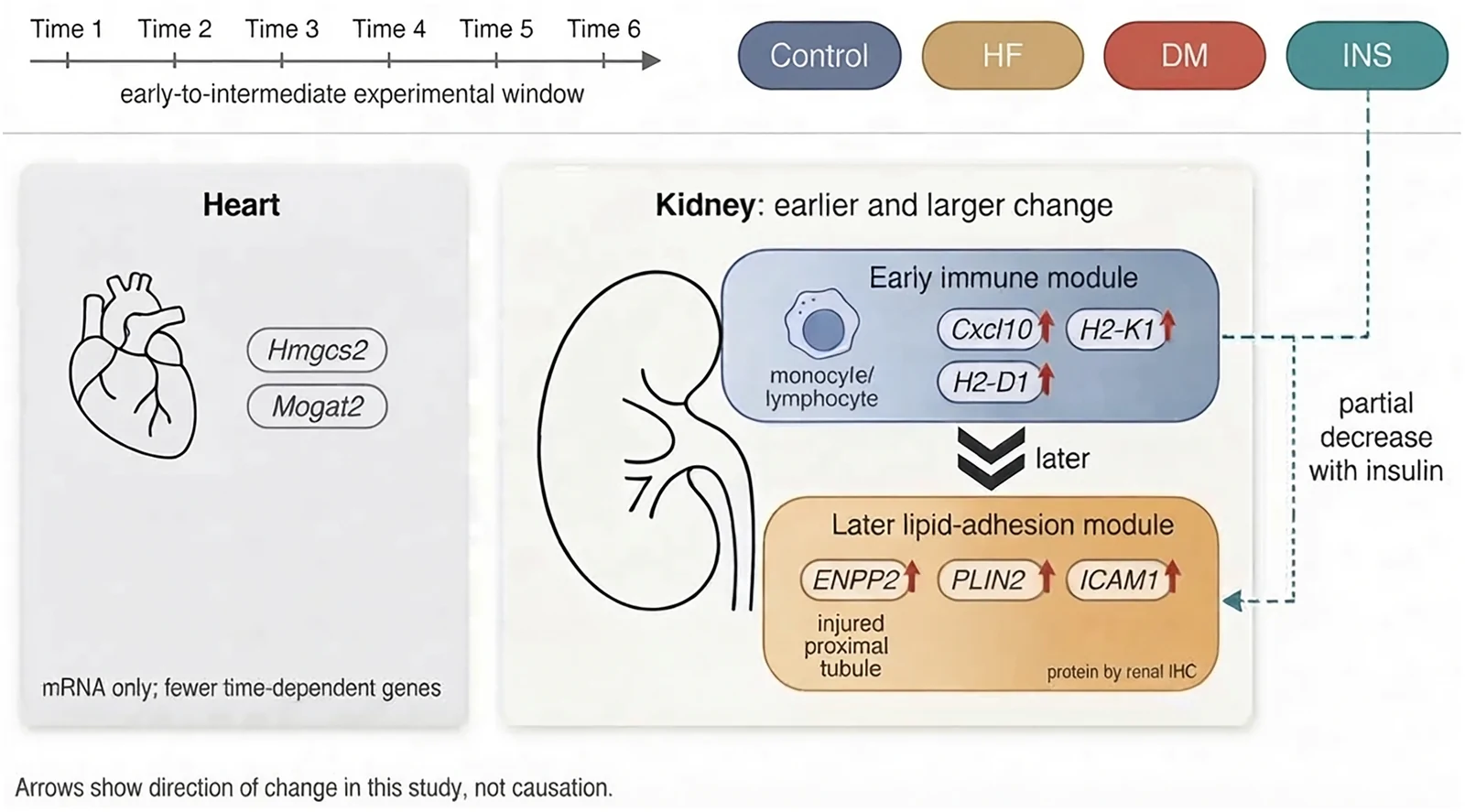
Time- and tissue-resolved changes supported by this study.

## Discussion

This study provides an exploratory characterization of continuous transcriptomic dynamics underlying diabetic cardiorenal complications. Conventional studies have largely relied on single-time-point analyses at fixed, often end-stage checkpoints to establish the molecular landscape of diabetic nephropathy (15). Bi-weekly sampling across the experimental window allowed us to track molecular changes during successive phases of metabolic stress. In this design, the kidney showed earlier and stronger transcriptomic divergence than the heart, adding a temporal dimension to diabetic tissue remodeling.

This time-based approach also revisits immune responses during diabetes progression. Many studies describe diabetic kidney disease as a steadily worsening inflammatory process linked to lipotoxic and immune injury (25, 26). In contrast, our time-series data revealed a biphasic immunometabolic pattern. Inflammatory and antigen-presentation genes such as Cxcl10, H2-D1, and H2-K1 increased strongly in the early phase (27) and then declined later in the sampling window. Although CXCL10 is often viewed as a persistent marker of renal inflammation and tubular injury, its expression here peaked early and decreased before fibrosis-related programs became dominant (28). These findings suggest that the kidney microenvironment can shift from acute immune activation toward chronic structural remodeling, a transition that single-time-point studies may miss.

Immunohistochemistry helped link selected transcriptional changes to renal tissue. Abnormal lipid accumulation under insulin resistance is an important cause of podocyte and tubular injury (29, 30). In our study, the lipid-droplet protein PLIN2 and the adhesion molecule ICAM1 increased markedly in the diabetic kidney. The rise in tubular PLIN2 is consistent with clinical observations linking tubular PLIN2 to faster kidney-function decline in DKD (31). PLIN2 promotes lipid-droplet accumulation and can impair mitochondrial respiration in tubular cells (32). Insulin treatment partially attenuated the increase in PLIN2, suggesting that glycemic control may ease lipotoxicity-related tubular stress. These protein data are kidney-specific: cardiac Mogat2 and Hmgcs2 were confirmed at the mRNA level only and were not assessed by immunohistochemistry.

High-fat diet alone caused clear ENPP2 upregulation in the kidney, without further increase in the diabetic state, suggesting that lipid excess and hyperglycemia may converge on shared fibrotic pathways. At this early stage, ICAM1 expression was lower in the HF group than in controls. This pattern differs from reports that high-fat diet and related metabolic stress activate NF-κB–driven renal inflammatory programs (33, 34) and may reflect sampling timing and model differences rather than a contradiction of those pathways. Immunohistochemistry localized ENPP2 mainly to injured proximal tubular cells (Inj.PT) in diabetic kidney, and insulin reduced this signal, consistent with the independent single-cell cohort and supporting cell-type-specific protein-level regulation. The proximal tubule is a major site of metabolic stress and lipid accumulation in diabetes. Consistent with this view, integrative lipidomics has linked disordered phosphatidylcholine metabolism through phospholipase A2 to CKD pathology and tubular injury (35). ENPP2-mediated LPA signaling from Inj.PT may promote local fibrosis and inflammation together with nearby fibroblasts and immune cells (36, 37). Beyond local LPA signaling, gut microbiota–derived metabolites have also been implicated in kidney disease progression and renal fibrosis and warrant longitudinal investigation (38, 39). Insulin partially reversed ENPP2 induction in our data.

Although independent qPCR and IHC were performed, larger cohorts will be needed. The discovery RNA-seq used one animal per group per time point, and only male mice were studied. Cardiac protein validation and cardiac histology were not obtained. In addition, the single-cell data came from a separate SGLT2 inhibitor cohort, which limits direct comparison with insulin and is not a head-to-head drug evaluation. Despite guideline-directed therapies that improve cardiorenal outcomes, residual renal risk persists in contemporary care (9–13); our map is therefore hypothesis-generating rather than a therapeutic efficacy study. From a translational perspective, recent work has highlighted metabolic biomarkers and therapeutic targets in CKD (40). Complementary literature also discusses DNA methylation and gut microbiota–derived metabolites as potential avenues for DKD intervention, including perspectives from traditional Chinese medicine research (41, 42); these ideas were not tested here. Future studies should include both sexes, paired functional measurements, and direct functional tests of ENPP2 and PLIN2, and should examine whether the lipid–inflammatory module identified here intersects with other metabolic modifiers.

## Conclusion

Diabetic metabolic stress reprogrammed the kidney earlier and more strongly than the heart. An early immune module was followed by later lipid and adhesion changes, including ENPP2, PLIN2, and ICAM1, in injured proximal tubule cells. Insulin was associated with partial modulation of selected trajectories. These results provide a time-ordered working map for experimental diabetic cardiorenal stress.

## Data Availability

The datasets presented in this study are available in online repositories. The raw sequencing data are available in the Genome Sequence Archive (GSA) under accession number CRA047585 at https://ngdc.cncb.ac.cn/gsa/browse/CRA047585. The public single-cell RNA-seq dataset is available in the Gene Expression Omnibus (GEO) under accession number GSE184652 at https://www.ncbi.nlm.nih.gov/geo/query/acc.cgi?acc=GSE184652. Additional supporting data are provided in the article and its **Supplementary Material**.

## Supplementary material

Supplementary Table 1 Sample information for bulk RNA-seq and sample sizes for RT-qPCR and immunohistochemistry. The workbook contains two worksheets: “bulk rna-seq” lists sample IDs, experimental groups, tissues, and time points; “IHC tissue” summarizes RT-qPCR and immunohistochemistry sample sizes by group and time point. File: Supplementary Table S1.xls.

Supplementary Table 2 Gene lists from the renal time-series analysis. Ensembl gene IDs and gene symbols are provided in paired columns labeled Control, HFvscontrol, DMvscontrol, and INSvscontrol. File: Supplementary Table S2.csv.
